# Cases of Fractures

**Published:** 1857-03

**Authors:** Frank Hastings Hamilton

**Affiliations:** Buffalo, N. Y.


// BUFFALO MEDICAL JOURNAL

AND

MONTHLY REVIEW.

**VOL. 12.** MARCH, 1857. **NO. 10.**

### ORIGINAL COMMUNICATIONS.

ART. I. — *Cases of Fractures.* Reported by FRANK HASTINGS HAMILTON, M. D., Buffalo, N. Y.

(Continued from Page 519.)

### TIBIA AND FIBULA.

### UPPER THIRD.

CASE I. — *Simple fracture. Result perfect.*

May 23, 1851, a man employed on a steamboat, broke both bones of his leg, below the knee. The tibia was broken just below the tubercle. He was about twenty-five years old. A German physician first examined the limb, but did not discover any fracture. On the eighth day he was seen by Dr. Mixer and myself. Motion and crepitus were then distinct, but the fragments of the tibia were not displaced. We applied a gutta percha splint, and he recovered in the usual time, and without deformity.

CASE II.—*Simple fracture. Result imperfect.*

Elias Tozer, æt. 21, fractured both bones of the leg about three or four inches below the knee. Drs. Beer and Singer, of Ohio, took charge of the case.

Five years after, I measured the limb and found it shortened, by over-riding of the fragments, five-eighths of an inch.

CASE III.—*Simple fracture. Result imperfect.*

Tim Logan, of New York. Simple fracture near the lower end of the upper third. He was treated at the New York City Hospital.

I saw him soon after union of the fragments had occurred. The leg was shortened half an inch.

CASE IV.—*Simple fracture. Delayed union. Result imperfect.*

F. C. Trask, of Erie Co., N. Y., æt. 35, broke his right leg in jumping from a buggy, in June, 1852. Fractures oblique in both bones: near the lower end of the upper third: simple. Drs. Sweetland and Aldrich in attendance.

The limb was dressed with lateral splints made of white wood, and with compresses and bandages, and then laid upon a pillow.

Eight weeks after the fracture had occurred, the gentlemen wished me to see the limb with them. I found Mr. Trask still in bed and the fragments not at all united.

Mr. Trask had enjoyed average health heretofore, but he was never very robust. When I was called to see him he looked pale; his skin was cold and moist; his pulse 120, and his appetite poor. The broken leg and foot were greatly swollen. The swelling was œdematous. Considerable excoriations existed on the back of the leg. The fragments were quite movable, and were overlapped three-quarters of an inch.

We agreed that the patient ought as soon as possible be got out of bed, so as to enable him to recover his strength, which had sadly declined. To this end a gutta percha splint was made to fit accurately the whole length of the leg; and having attached a large number of tapes, it was to be secured upon the limb. Several times each day it was to be removed, and the limb bathed with brandy and water. Slowly also the limb was to be brought down to the floor and the patient be made to sit up, and as soon as possible he was to walk with crutches, or to ride.

Nov. 4, 1852, Mr. Trask visited me at Buffalo. The directions had been followed implicitly. About two weeks after my visit, he rode out, and in about nine weeks, or seventeen weeks from the time of the fracture, the bones were found united. His health and strength were quite restored, and the limb was no longer œdematous. It is straight, or with only a slight projection of the upper fragment in front of the lower, and it is shortened three-quarters of an inch.

Case V. — *Simple fracture. Result imperfect.*

James Kelly, æt. 39. Simple fracture of right leg, between three and four inches below the knee joint.

The limb was dressed by a surgeon in Ireland.

Six years after, Nov. 7, 1858, I found the limb shortened one inch and a half, and much bent backward at the seat of fracture. Knee partly anchylosed.

Case VI. — *Compound fracture. Result uncertain.*

Frank Goodrich, of Buffalo, aged about 30 years, broke his left leg near the lower end of the upper third. The integuments were penetrated and much bruised.

I was called on the same day, Aug. 25, 1847, and proceeded immediately to dress the fractures. The fragments were easily replaced, but they did not support each other sufficiently to prevent a shortening when allowed to take care of themselves.

I dressed the wounds in a simple manner, and then applied first a flannel roller, and over this felt splints, secured with additional turns of the roller. The limb was laid over Post's double inclined plane, and so secured to the foot piece as to make moderate extension..

On the fortieth day all dressings were removed, and the limb was firm, straight and of the same length as the other. The other leg had been broken some years before. (See Case VII.)

Case VII. — *Simple, comminuted fracture. Result uncertain.*

Frank Goodrich, aged about 25 years, was thrown from a horse, breaking his right leg four inches below the knee, near the middle, and again near the ankle. The fracture was treated by Drs. Snow and Burwell, of Buffalo.

Five years after I find the limb quite straight, but the several points at which he says the fractures occurred, are tender, and present irregularities, which confirm the patient's statements. It is probable that the limb is shortened, but as the other leg was broken when I examined this leg, I could not determine. (See Case VI.)

Case VIII.— *Compound fracture. Result imperfect.*

Joseph Roach, æt. 34, broke his leg near the lower end of the upper third. Dr. Prentice, of Lagrange, N. Y., was employed. Fracture compound.

Eight years after the date of the accident, the leg was shortened three-quarters of an inch, and very much bent at the seat of fracture.

Case IX.— *Compound, comminuted fracture. Result imperfect.*

Rossel B. Davis, of Versailles, Cattaraugus Co., N. Y., æt. 50. June 1, 1854, he was kicked by his horse while sitting in his wagon, and immediately thrown out.

Drs. Ellis and Babcock were immediately called, and dressed the limb.

June 2. I met these gentlemen in consultation. I found the tibia had been broken four inches below the joint. We could not determine where the fibula was broken, but believed it to be near the same point. Dr. Ellis had removed five fragments of bone, and had left other fragments whose attachments to the soft parts underneath rendered their removal difficult. They then laid the limb upon a well cushioned board, and applied a many-tailed bandage and side splints. It had bled constantly for twenty-four hours, from two wounds, each of which seemed to be made by the corks of the horse's shoe.

I made very little change in the treatment except to place the limb in a box. Cool water dressings were directed, and after a few days it was advised to fill the box with bran.

At the end of four weeks there was no union and the leg was shortened one inch. Extension was then applied, and July 20 the starch bandage was added.

Aug. 6, tolerably firm union had occurred, and the limb was shortened only three-quarters of an inch. Since then several fragments of bone have escaped.

April 1, 1855, he was about his farm.

Oct. 6, 1856. He still walks with a slight halt.

Case X.—*Compound fracture, complicated with fracture of the opposite femur. Death in fifty-four hours from the shock.* (*See Case* LXXXVII *of Fractures of Femur.*)

Catherine Henriger, æt. 68, jumped from the second story of a dwelling house, March 15, 1853, breaking the left femur about its middle, and the right leg near the lower end of the upper third.

She was taken to the hospital and placed under my care. She died in fifty-four hours, having never recovered from the shock.

### MIDDLE THIRD.

Case XI.—*Simple fracture. Result perfect.*

Walter Redmond, of Buffalo, æt. 38, was admitted to the Buffalo Charity Hospital, with a fracture of both bones of the leg a little below the middle, Dec. 15, 1851. Fracture occurred three days before admission, in consequence of a fall upon the sidewalk.

I applied a paste bandage, on the 17th of Dec., and Jan. 17th, 1852, I removed all dressings. The fragments were united without either shortening or perceptible deformity. A small amount of ensheathing callus at the seat of fracture.

Case XII.—*Simple fracture. Result perfect.*

John Maloney, æt. 16, was admitted to the Buffalo Hospital with a fracture of both bones of the leg near the middle. Fracture simple. Bones never displaced.

It united in the usual time without shortening or deformity.

Case XIII.—*Simple fracture. Result perfect.*

Thomas Cassody, æt. 17, admitted to the Buffalo Hospital, Oct. 7, 1853, with an oblique, simple fracture of the left leg, in its middle third. The accident occurred the day before admission, from a blow with a piece of wood. It was dressed by a physician in town with a roller and side splints.

Oct. 8, I removed the dressings and laid the limb over a double inclined plane, making some extension by means of a paste gaitre. The limb was swollen at the seat of fracture.

Oct. 9. Opened the dressings.

Oct. 16. Renewed the dressings. No union. No ensheathing callus.

Oct. 19, removed limb from double inclined plane, applied paste bandage.

Nov. 7. Union incomplete. Removed all dressings. Laid limb on a pillow and directed cold water baths.

Nov. 17. Union complete. No ensheathing callus. No shortening or deformity.

Case XIV.—*Simple fracture. Result perfect.*

Edward Evans, of Buffalo, æt. 10, fractured the left leg near its middle. Dr. Chapin, of Buffalo, was employed.

Fifteen years after the accident occurred, the limb was perfect.

Case XV.—*Simple fracture. Result perfect.*

Wm. McCormick, of Buffalo, æt. 19, broke both tibia and fibula near their middle. Fracture simple and transverse.

I applied a paste roller. The fragments united without shortening or deformity.

Case XVI.—*Simple fracture. Result perfect.*

Joseph Werner, æt. 15, was caught under a load of wood which was capsized, Sept. 24, 1852, breaking the left tibia obliquely near its middle. Crepitus and motion distinct. I think the fibula was broken also, but I cannot say positively, since I could not feel any crepitus or motion which could be clearly charged to it. The general mobility of the limb, however, and the severity of the injury, seemed to imply a fracture of the fibula.

I dressed the limb with a gutta percha splint, and laid it upon a double inclined plane.

On the 16th day motion was distinct between the fragments. Removed double inclined plane, under the belief that the position increased the liability to motion between the fragments. Continued side splints, and laid the leg on a pillow.

On the 21st day union had occurred, but the limb was slightly bent at seat of fracture. The fragments were displaced outward. As the callus was still flexible, I laid the limb again upon the double inclined plane, and used some force to straighten the fragments. This procedure was successful, and did not hurt him but very little.

On the 27th day, in presence of Dr. Bowen, I removed all dressings. No shortening; no deformity; very slight ensheathing callus. Reäpplied side splints only.

Case XVII.— *Simple fracture. Result perfect.*

John Stancleft, of Buffalo, æt. 20, was struck by the boom of his vessel breaking both bones of one leg, near the lower end of the middle third. The accident occurred Nov. 12, 1854, and he was brought to the hospital and placed under the care of Dr. Wilcox, marine surgeon, Nov. 13.

I saw him repeatedly during the progress of the cure. The fragments were united on the twenty-second day, and I could not discover, at any time ensheathing callus.

Oct. 18, 1856. The limb was neither shortened, nor in any manner deformed. The ankle, however, was occasionally painful.

Case XVIII.— *Simple fracture. Result perfect.*

Thomas Ryan, æt. 31. While walking on the railroad track, Jan. 28, 1854, he slipped and fell, the back of his right leg coming upon the iron rail, and breaking it at a point, I think, rather below the middle. The fracture was simple.

On the following day he was brought to the hospital, the limb not having yet been seen by a surgeon, or dressed. I found the fragments not displaced, and the limb very little swollen.

We laid the limb upon a pillow, and having f lded the pillow around its sides, we laid along, outside of the pillow, two wooden splints, and these were secured in places by strips of bandage tied over the pillow and splints together. The foot was also carefully supported.

On the fourth day, Dr. Pupikofer, the intelligent house surgeon, applied a paste bandage to the whole limb.

March 10th, six weeks from the date of the fracture, all dressings were laid off. The bones were firmly united, and without ensheathing callus. The limb was neither shortened nor deformed.

CASE XIX.— *Simple fracture. Result perfect, according to my notes; but subsequently found by another surgeon to be shortened, &c.*

Joseph Beyer, æt. 70. A heavy stone fell against his left leg, Aug. 30, 1854, breaking both bones near the middle. Fracture simple.

I do not know who had charge of him at first, but on the eighth day he was brought to the Buffalo Hospital, during the service of Dr. Chas. Winne. Beyer says the fragments were never displaced. Side splints, &c., were applied.

Oct. 1, 1854, when I took charge of the limb, four weeks and five days after the occurrence of the fracture, bony union was complete, and I removed all dressings. On measuring the limb I could not detect any shortening, nor could I discover any displacement of the fragments. I find, however, a note made subsequently by the house surgeon, to the effect that the limb was shortened one quarter of an inch, and that the lower fragment was advanced slightly in front of the upper, so as to interrupt the anterior line of the spine of the tibia. There was no ensheathing callus, he also adds.

CASE XX.— *Simple fracture. Result perfect, except that an ulcer exists over the seat of fracture.*

Michael Gallaghar, æt. 63, fell through a hole in the floor, breaking his leg near the lower end of the middle third. Dr. Weyburn, of Detroit, was employed. In four months he was able to walk.

Jan. 1856, about twenty months after the accident, he was received into the Buffalo Hospital, for an irritable and large ulcer, situated over the point of fracture. He says that during the progress of the cure a blood blister formed over the fracture, and that it soon formed an ulcer, which has never healed.

The limb is neither shortened nor deformed.

CASE XXI.— *Simple fracture. Result perfect.*

Henry Simpson, of Buffalo, æt. 30, was kicked violently by a man with whom he was wrestling, breaking the tibia and fibula of the left leg near the lower end of the middle third. This occurred April 27th, 1853, and I was immediately called. The limb was already a good deal swollen over the seat of fracture, but I was able to detect crepitus and motion in both the tibia and fibula; there was, however, no perceptible displacement of the tibia.

I applied my lateral splints, and laid the limb upon a pillow.

May 13, I examined the limb and found the fragments not yet united. No ensheathing callus. A paste bandage was substituted for the side splints.

June 2. The paste bandage was opened. Union of fragments complete. No ensheathing callus, and the limb not shortened or in any manner deformed.

Case XXII.—*Simple fracture. Result perfect.*

John Heisler, 151 Oak street, Buffalo, æt. 14, fell on the sidewalk, Jan. 13, 1854, breaking his left leg six inches above the ankle. The fracture was simple, and the tibia was broken transversely; fragments not displaced.

I dressed the limb with a paste bandage.

Feb. 22, the bones were united and I could not detect any ensheathing callus. The limb was in all respects perfect.

Case XXIII.—*Simple fracture from muscular action. Result perfect.*

F. Young, æt. 41, fell in March, 1855, while walking upon a slippery sidewalk, breaking the left leg near its middle. He thinks the leg broke before he struck the ground. Fracture simple and oblique.

Dr. Nelson, of Buffalo, was called, and dressed the limb with moulded copper side splints, laying the limb upon its side. On the third day Dr. Nelson requested me to see the patient with him. The fragments were then in place, nor had they ever been much displaced; yet motion and crepitus were distinct. The treatment was not changed. Three months later, the limb was well and in all respects as before.

Case XXIV.—*Simple fracture. Result imperfect.*

Wm. Baker, æt. 38, fractured his leg in Sept., 1852, and was taken to the Erie County Alms House. His health was good previous to the accident. The fracture was simple, and in the middle third. Drs. Forbush and Winne had charge of the limb.

Eight months after the accident occurred, he was unable to walk except with crutches, owing to the swelling and soreness about the ankle-joint and foot. The leg was shortened half an inch. The upper end of the lower fragment of the tibia now projects forward slightly and over-rides the lower end of the upper fragment.

During a part of the time it had been treated with the paste bandage.

Case XXV.—*Simple fracture. Result imperfect.*

Mary Jane Carney, æt. 18. Admitted to the Hospital of the Sisters of Charity—Dr. Charles Winne's service—Sept. 19, 1853, with a fracture of the leg in its middle third.. Fracture simple and oblique. The accident had occurred two days before by being thrown down upon the sidewalk. Dr. Root, of Buffalo, had first dressed the limb.

Oct. 1, when I took charge of the ward, the limb had been dressed only two days before, and I saw no occasion to disturb it.

Oct. 8, I removed the dressings and found the limb shortened three-quarters of an inch. Fragments partially united. No ensheathing callus. I dressed the limb with a paste bandage and with splints of pasteboard.

Oct. 18. Opened the dressings. Union not complete. No ensheathing callus.

Nov. 6. Removed dressings entirely. Union complete. Shortening same as when I first took charge of the case. The upper fragment was a little in front of the lower. No ensheathing callus. I permitted her now to walk with crutches.

Case XXVI.—*Simple fracture. Result imperfect.*

J. C., of Buffalo, aged about 25 years, had his leg broken by the falling of a heavy plank upon it, Feb. 25, 1854. The fracture in the tibia was oblique and about six inches above the ankle. The fibula was also certainly broken, but at what point, I could not determine. The plank struck also upon the back of his neck and produced a concussion of the spine, which paralized the bladder, and for several days I was obliged to relieve his bladder with a catheter.

I dressed the limb with junks and side splints, and laid it in a box.

March 14th, seventeen days after the accident occurred, there was no union of the bones, no ensheathing callus, very little swelling, and no displacement. From this time he remained under the care of another surgeon until nearly the eighth week. He was then taken to the hospital, where he remained three weeks longer.

July 31, 1854, I found the limb shortened one inch, but with very little deformity. He wears an extra heel under this foot.

Case XXVII.— *Simple fracture. Result imperfect.*

Honore More, æt. 18, admitted to the Buffalo Hospital of the Sisters of Charity, June 18, 1853. Dr. Charles Winne's service. Simple, oblique fracture of right leg near the lower end of the middle third. Considerable inflammation and swelling followed. Dressed with Scultetus bandage and side splints.

Fourteen weeks after, at the beginning of my service, I found the leg shortened one inch; the upper fragment overlapping the lower and lying upon its outer, or fibular side. The foot still remained swollen and she was able to walk only with crutches. I ordered the bandages to be removed, and directed the limb to be rubbed with a stimulating liniment.

Nov. 6, five months from the time of the accident, she was just beginning to walk without crutches. At no time, after she came under my charge, could I detect ensheathing callus.

Case XXVIII.— *Compound fracture. Result perfect, except that a fistulous discharge has continued from the point of fracture.*

The infant daughter of Mr. Armstrong, residing on Washington street, had her left leg broken, Sept. 9, 1852, a little below its middle. A woman had fallen upon the leg accidentally, and the broken tibia was thrust through the skin.

Dr. Samo was called, and, by his request, I saw the patient with him. We found the upper fragment of the tibia projecting through the skin, and at first we were unable to reduce it. We then gave her chloroform, and with great effort finally succeeded. We dressed the limb with a gutta percha splint, and laid it upon a pillow.

Oct. 21, six weeks after the accident occurred, I found the limb firmly united and straight. Some ensheathing callus could be distinctly felt, and a fistulous discharge continued from the seat of fracture.

Case XXIX.— *Compound fracture. Result perfect.*

Wm. Kearney, æt. 35, fractured both bones of his leg near its middle. Fracture compound. Dr. Leavenworth, of Cattaraugus, N. Y., was employed

Four months after, I found the bones united without displacement, and the wound healed.

CASE XXX.— *Compound fracture. Result nearly perfect.*

Mrs. Margaret Conner, æt. 65, was admitted to the Buffalo Hospital of the Sisters of Charity, Dec. 19, 1856, with a compound fracture of the left leg. Fracture at the lower end of the middle third, very oblique. Direction of fracture downward and inward: the upper fragment being in front of the lower. The accident had resulted from a fall upon the ice, four days before her admission, and the bone had been thrust entirely through the skin. It was dressed by Dr. Garvin. When admitted, the limb was much swollen and vesicated.

I dressed the limb with gutta percha splints carefully moulded, and laid it upon its outside, after the method of Pott.

Jan. 1, 1857, finding that she would not lie upon her side, and that the limb was liable to displacement on account of her frequent changes of position, I laid her leg over Day's double inclined plane, and secured it snugly.

Jan. 14th. Union firm, with a shortening of one-quarter of an inch. Removed the leg from the double inclined plane.

Feb. 7th. The limb looks and feels perfectly straight, and one cannot discover that it is shortened, except by measurement.

CASE XXXI.— *Compound fracture. Result imperfect. Delayed union. Slough on heel, &c.*

W. S. Mooney, æt. 35; a farmer. In the winter of 1853 and '54, both legs were caught in a threshing machine, breaking the right leg near the lower end of the upper third, and the left leg near the ankle.

Dr. Ely dressed his limbs and continued in charge to the close of the treatment. The legs were both laid over a double inclined plane. The wounds occasioned by the injury were not healed at the end of seven weeks, nor was the bone united. There was also at this time a large slough upon the back of the heel.

About the first of May, 1854, four months after the injury was received, he began to walk upon crutches.

Jan. 2, 1855, one year after the accident occurred, I examined his leg at the Buffalo Hospital.

His right leg was quite crooked, the lower part of the limb having fallen back out of line. Mooney says this was occasioned by a pillow which was laid under the fracture to prevent pressure upon the heel. The leg is shortened three-quarters of an inch.

CASE XXXII.— *Compound fracture. Result imperfect.*

John Phaley, æt. 25. Fractured at the same time his right arm, and left leg—also dislocated right shoulder. (Fractures of humerus, Case XXXIV.) Fracture of leg compound. I think it was broken below the middle.

Dr. Rogers, of Dunkirk, first dressed the limb. On the third day it was dressed by Dr. Pratt, of Buffalo.

On the twelfth day he came under my charge at the hospital. The fragments were already quite firm. Leg shortened one quarter of an inch. The same was the condition of the limb when he left the hospital. No extending apparatus was ever employed.

CASE XXXIII.— *Compound fracture. Result imperfect.*

Thurstone Carpenter, æt. 4, received an injury, breaking both bones of the leg near its middle. Fracture compound. It was treated by Dr. Chapin, who was at that time, and for many years, reputed the most skillful surgeon in Buffalo.

Twenty-three years after the accident, Mr. Thurstone called upon me on account of a paralysis of his lower extremities, which had recently occurred. He stated, that from the time of the fracture until within about one year, an open ulcer had existed over the seat of fracture; and that soon after it had closed over completely he began to loose the use of his limbs. During the time it was open, small scales of bone have frequently been thrown off. The limb is half an inch shorter than the other.

I advised a renewal of the ulcer.

CASE XXXIV.— *Compound fracture. Result imperfect. Abscess and ulcer.*

Joseph McEllicott, æt. 22. Fracture near the middle of the left leg. Compound and oblique. At the same time also he fractured his right thigh and his lower jaw. (See Fractures of Femur, Case LXXIII.)

He was admitted into the Buffalo Charity Hospital three months after the fracture was received. The bones of the leg were then united, but with a shortening of an inch and a half; the upper fragment riding in front of the lower. The wound over the seat of fracture was not healed, and there was an abscess in the back part of the leg, which still continued to discharge pus freely. On the back of the heel there existed also a deep ulcer.

CASS XXXV.— *Compound fracture. Death.*

Martin Foot, of Pittsfield, Mass., æt. 8, had his right leg broken in a cider mill, Oct. 22, 1841. Fracture of tibia transverse, at a point a little below the middle of the leg, and the end of one fragment was thrust four inches through the skin, being accompanied with extensive laceration.

I reduced the fragments and closed the wounds with sutures and adhesive plasters, applying also one side splint, with Scultetus bandage. The limb was laid upon a pillow, elevated, and cold water dressings directed.

I did not see this patient again, but he was left in the charge of Dr. Henry Childs, of Pittsfield, Mass., from whom I subsequently learned that the lad survived the accident only two or three months.

CASE XXXVI.— *Comminuted fracture. Both legs broken. Result uncertain.*

Michael Carmody, æt. 19. May 9, 1849, a heavy bar of iron fell across his legs, breaking the right tibia and fibula, four inches above the ankle, and again near the middle. Both fractures of the tibia oblique. The left tibia and fibula were also broken, just above the ankle. Michael was received at the hospital on the same day, and both legs were at once dressed with dry rollers, and supported with splints and pillows.

May 16, seven days after the fractures, the swelling of the limbs being considerably reduced, the starch bandage of M. Suetin was applied to each leg.

May 18, he was allowed to sit up in bed or in a chair.

May 29, the swelling having still more subsided, the bandages were found slightly loosened. They were laid open and made smaller by cutting out a small strip, from the knee to the toes, and afterward reädjusted and secured by a dry roller.

June 13, five weeks after the fracture, the dressings were removed, and the limbs were sound and seemed perfect, except that considerable stiffness existed in the left ankle-joint. As both legs were broken, however, it is impossible to say positively that they were not shortened.

CASE XXXVII.— *Compound, comminuted fracture. Result perfect.*

Luther C. Peck, Nunda, Livingstone Co., N. Y., æt. 14, was run over by a loaded sleigh, breaking his left leg through its middle third. The fracture was compound and comminuted. It was treated by Dr. Thayer, of Onondaga Hill, N. Y. No extension was employed.

After forty years, I examined the limb and could find no trace of the injury.

CASE XXXVIII.— *Compound, comminuted fracture. Union with shortening.*

Isaac Hoffer, of Buffalo, æt. 28, was thrown from a wagon, June 16, 1845, breaking the tibia and fibula of the left leg. The fracture was oblique, compound and comminuted, a fragment half an inch in length being removed immediately. The fracture occurred a little below the middle of the leg.

The limb was dressed by Drs. Sprague and Bronck, assisted by myself, with side splints, and then laid upon a double inclined plane.

On the seventh week the external wound had closed and the bones were united. I then removed the splints and applied a paste bandage. This also was removed on the fifty-seventh day.

The leg is shortened half an inch.

CASE XXXIX.— *Compound fracture. Result imperfect.*

Jeremiah Caveney, æt. 33. Oct. 8, 1855, his right leg was caught between the side of a railroad car and a water tank, producing a compound fracture near the lower end of the middle third. Fracture of the tibia nearly transverse.

Dr. Charles Winne, assisted by Dr. E. P. Smith, saw and dressed the fracture temporarily, about six hours after it occurred. A single splint was applied to the outside of the leg, which was retai ed in place by a roller; he was then taken to the Hospital of the Sisters of Charity: the limb was laid upon a pillow, and cool water lotions applied.

Oct. 9, I found him at the hospital. The bleeding had nearly or quite ceased. The circulation in the limb being rather imperfect, I suspended the cool water lotions, but left the dressings undisturbed.

Oct. 10. Cut open the dressings opposite the fracture, but did not disturb the splint, still fearing that the bleeding might recur. Wound in the flesh gaping, and four and a-half inches in length. The tibia could be seen through the wound. I was unable to bring the edges of the wound together; I therefore laid over it a piece of lint spread with cerate.

Oct. 12. The limb had become more swollen and inflamed. He did not complain of the dressings, but on removing the splint we found it had produced an ulceration over the malleolus externus; cool water irrigations were again ordered, the limb being carefully supported by a pillow covered with an oil cloth.

Oct. 14. Swelling and inflammation were still increasing. The limb would not bear any pressure even such as was necessary to keep the fragments in place. I had it suspended carefully in a sacking, after the manner of Mayor, and continued the irrigations.

Oct. 19. We found it impossible to steady the limb in this position, and it was transferred to a box of bran, into which it was carefully and snugly packed. Still, however, we were unable, by any amount of pressure which he could tolerate, to prevent the lower end of the upper fragment from tilting forward. This inclination had been noticed from the first, but never successfully resisted. The swelling had now begun to subside.

Oct. 27. The wound was discharging freely; the swelling was abated, but he could not bear any pressure, not for a moment; nor could he tolerate any efforts on our part to restore the fragments.

Nov. 13th, the limb was taken from the box.

Dec. 31. It is on this day first mentioned in my hospital records, that the fragments had united firmly. I think, however, it was united some time before. The leg had shortened only about one-quarter of an inch, but the upper fragment had slid forward upon the lower fragment nearly the whole of its diameter, making a remarkable projection in front. The lower part of the leg and foot fell slightly backward. It was five months before he began to walk without crutches.

Aug. 24, 1856, nearly three years after the accident, I examined the limb and found the same shortening of the limb and the same deformity, but the limb as useful as the other. There is no ulceration, but only some unusual tenderness over the projecting bone. Over the malleolus externus is seen a scar about one inch in diameter, left by the ulceration occasioned by the splint. There is no stiffness in the ankle and he walks firmly and without the slightest halt. He is a laborer, working day and night through a great part of each summer, in a wheat elevator, and receiving wages of $36 per month. He says he is entirely satisfied; he knows that every possible care was taken to prevent deformity, and he is thankful that his limb was saved at all.

I have related this case unusually at length, because it illustrates what great deformity may sometimes result where the utmost care and attention are employed in the treatment; and also how great may be the deformity in other cases, without any degree of maiming.

Case XL.— *Compound, comminuted fracture. Result imperfect.*

John Ott, of Buffalo, æt. 48, was admitted to the hospital, Dec. 18, 1849, with a compound, comminuted fracture of the left leg. He was also much bruised about his back, and his scrotum was badly lacerated. A wagon load of wood had fallen upon him.

We simply supported the limb in a box until the eighth day, when, the swelling having sufficiently subsided, we applied a paste bandage, and also made extension and counter-extension over a double inclined plane.

At the end of five weeks we removed the limb from the splint and found the bones united with a shortening of half an inch. During the treatment we had used all the extension he could bear, and, indeed, we had frequently to relax the extension. We had also the usual difficulty where the limb is laid upon its heel of more or less pain in this part, compelling us to make frequent changes.

Nine months after the fracture occurred, he still complained of a stiffness in his ankle and knee-joint.

Case XLI.— *Compound, comminuted fracture. Both legs. Result imperfect.*

John Curran, æt. 23. Both legs broken by the fall of a heavy stone upon them. Right leg broken about its middle. Comminuted. Anterior tibial artery ruptured. Left leg broken in a similar manner near the ankle.

I laid both limbs over a double inclined plane made of pillows, covered with oil cloth. To both legs I applied light side splints. The limbs were elevated and kept moist with cool water dressings. In the afternoon of the same day, the swelling had greatly increased, and on the sixth day I placed the right leg in a box containing bran, and the left in a swing splint. No suppuration or sloughing of consequence occurred, although the left leg was very cold and insensible during several days, and I was obliged, after the first day or two, to let it rest upon a lower plane, and to discontinue the cold water.

At the end of two weeks I applied, for the first time, moderate extension and counter-extension. After four weeks all extension was laid aside, the bones having united with a shortening of about three-quarters of an inch in each leg, and with corresponding deformity.

It is quite probable that if we had made any attempts to extend either limb on the first few days, it would have resulted in the loss of both.

CASE XLII.— *Compound, comminuted fracture. Death on the fifth day.*

John Harkner, of Buffalo, æt. 20, was struck by a falling tree, Jan. 30th, 1852, breaking his right leg near its middle. The skin and flesh were extensively torn, and both bones were broken and slightly comminuted. Dr. Bryant Burwell and myself, in attendance.

In order to reduce the bones I was obliged to enlarge the opening in the skin. I tied the anterior tibial artery, which was wounded and exposed. Finding that I could not retain the fragments of the tibia in place, I bored their ends with a shoemaker's awl and wired them together. I dressed the limb carefully and laid it in a well moulded gutta percha splint, supporting it with pillows, &c.

Harkner died on the fifth day from a low irritative fever, supervening upon the shock.

CASES XLIII AND XLIV.— *Compound, comminuted fracture, complicated with extensive laceration. Amputation and death.*

Thomas Russel, æt. 20, was run over by a railroad car, Jan. 16, 1853, crushing both legs through their middle thirds. I amputated the right leg, three inches below the knee, sixteen hours after the accident occurred, and the left leg twenty-four hours after the accident. He survived the last operation forty-eight hours, having never completely rallied from the shock of the injury.

CASE XLV.— *Compound fracture, complicated with a fracture of the femur on the same side. Result imperfect.* (*See Case C. of fractures of femur.*)

Thomas Dolanty, of Buffalo, æt. 31, was run over by a loaded cart, Aug.

14, 1854, breaking his right thigh about six inches above the knee, and his leg eight inches above the ankle. I applied a straight splint with extension, using a paste gaiter to secure the leg and foot to the foot piece. I also applied bran junks along the leg and thigh. On the tenth day, at my request, Dr. Boardman took charge of him.

At the end of seven weeks the fragments were united, but overlapped one quarter of an inch. The point of overlapping was very perceptible. There was no appearance of ensheathing callus. The limb was straight.

Case XLVI. — *Compound, comminuted fracture. Amputation on thirteenth day. Death on the sixteenth day.*

John Slack, of Buffalo, about 40 years old, had his right leg broken, June 28, 1847, by being caught between the buffers of a couple of railroad cars. Dr. White, of Buffalo, in attendance.

Dr. White requested me to see the patient with him. Probably three inches of each bone were completely comminuted, being broken into fifteen or twenty fragments. These fragments were mostly removed with forceps. Considerable venous hæmorrhage existed, but the arterial circulation in the foot was unimpaired. Both the anterior and posterior tibial arteries were pulsating distinctly. The constitutional shock was not excessive.

We determined to attempt to save the limb. The limb was accordingly laid in a box well lined with cotton batting. Cold water dressings were applied to the wounds.

On the ninth day he had a slight diarrhœa, but the red line of demarcation was established around all the sloughs.

On the thirteenth day he was more feeble, and the wounds looked unhealthy. By advice of Drs. Josiah Trowbridge, Scott, Wilcox, and myself, Dr. White also concurring, Dr. White amputated the limb six inches above the knee. On the sixteenth day after the accident, and three days after the amputation, he died.

Case XLVII. — *Compound, comminuted fracture. Result imperfect. Prosecution and successful defence.*

Wm. Hunter, of Cayuga Co., N. Y., was caught in a steam engine, sometime in Sept., 1848, breaking and comminuting his leg near the lower end of the middle third. Dr. S. H. Plumb, of Sterling, Cayuga Co., N. Y., a young and promising surgeon, was employed.

Subsequently, the man refusing to pay, Dr. Plumb sued for his account in a Justice's Ccurt, and Mr. Hunter was notified of the fact, but did not appear to defend, although he was in town and could have done so. Dr. Plumb having proved by competent witnesses the amount and value of his services, a judgment was entered by the Justice against Hunter.

In May, 1855, Hunter brought an action against Dr. Plumb, in the Cayuga Co. Circuit Court, Hon. Judge Johnson, presiding, for malpractice; on the part of the plaintiff, Messrs. George W. Rathbone and Warren T. Worden, of Auburn, appeared as attorneys: and on the part of the defendant, Messrs. A. G. Hull, of Fulton, Oswego Co., and D. Gott, of Syracuse.

The testimony on the part of the plaintiff having closed, Mr. Hull moved a nonsuit, on the ground that this case had already been tried before a Justice, and this constituted a bar to any further action. After considerable discussion on the part of the opposing attorneys, the Hon. Judge decided to sustain the motion for a nonsuit, and the case was dismissed.

An appeal was obtained from this decision, but it was affirmed at the General Term.

It was the wish of Dr. Plumb that the question should have been tried upon its merits, and that it should have gone to the jury, since the testimony offered by the plaintiff himself fully acquitted the defendant of malpractice. The limb had been carefully and skillfully managed by Dr. Plumb: being dressed with rollers, side splints, junks, &c., and, during most of the time, supported in a box; and notwithstanding the severity of the original injury, and the violence of the inflammation which followed, the limb was shortened only three-quarters of an inch. It was also slightly bent at the seat of fracture. The limb was, however, as useful as before.

I think Hunter was 32 years old, and at the time of the trial I saw him walking about briskly and without any perceptible halt.

### LOWER THIRD.

CASE XLVIII.—*Simple fracture from muscular action. Result perfect.*

Dr. Josiah Trowbridge, of Buffalo, æt. 42, slipped upon the sidewalk, and fell, breaking the left tibia and fibula about three inches above the ankle. The bones broke in the effort to sustain himself, for he heard them snap before he reached the ground. Dr. Trowbridge was a very healthy, but rather heavy and muscular man. The fragments were not seen to be displaced.

Drs. Marshall and Stagg, of Buffalo, dressed the limb with a carved splint, and without extension. In four weeks he walked on crutches. He never saw or felt the smallest amount of ensheathing callus upon the tibia.

Twenty-four years after, April, 1852, when I examined his leg, no traces of the fracture could be seen.

CASE XLIX.—*Simple fracture from muscular action. Result perfect.*

Mrs. J. P. W., of Buffalo, aged about twenty-five years. Mrs. W. weighs nearly two hundred pounds. Jan. 10, 1852, she was descending her door steps with an infant in her arms, when, the steps being covered with ice, she slipped and fell, breaking her right leg just above the ankle. Mrs. W. says she felt and heard the bone snap before she touched the steps. Of this she is certain.

Dr. Flint and myself in attendance.

I found the tibia broken obliquely, the fragments being quite movable, but not much, if at all, displaced. The limb was dressed with a carefully moulded and well padded gutta percha splint, and then laid in a pillow upon the bed. Mrs. W. experienced unusual pain from the fracture, for several days, for the relief of which I was compelled at times to permit her to inhale chloroform. She was of a nervous temperament, and had frequently resorted to chloroform before to relieve neuralgic pains. The limb became very much swollen and remained so for a week or two. No extension was ever employed.

Within the usual time the bones united without shortening or deformity, and in about four months she was able to walk without any halt.

CASE L.—*Simple fracture. Result perfect.*

John C. Crooks, æt. 14, fractured his right leg in its lower third. Fracture oblique. Dr. Jewett took charge of the case.

Eight years after, while Mr. Crooks was studying medicine, I examined the limb and found it perfect.

CASE LI.—*Simple fracture. Result perfect.*

Wm. A. Ford, of Buffalo, æt. 34. Mr. Ford, who weighs about one hundred and forty pounds, jumped from the driver's seat of a coach, holding in his hands a satchel weighing about thirty pounds, striking square on the soles of his feet, upon a stone pavement. I was immediately called, (Oct.

23, 1853,) and found his right leg broken through its lower third; the tibia being broken nearly transversely, four inches above the ankle, and the fragments not displaced. The fibula was broken three inches above the joint, and the lower fragment inclined toward the tibia, while the upper fragment was in place. Over the lower end of the upper fragment of the fibula was a large swelling.

Assisted by Dr. Pupikofer, I applied pasteboard splints and bandages.

The fractures united readily in the same position in which I at first found them. There was no shortening or deformity at the end of one year after the fracture occurred.

Case LII.—*Simple fracture. Result perfect.*

Robert Collinswood, of Buffalo, æt. 44, fell down three steps, Aug. 2d, 1856, breaking the right leg about four inches above the ankle. Tibia broken, obliquely, downward, and to the tibial side. Simple. Mr. C. weighed at this time, 196 lbs.

I found the fragments of the tibia only very slightly displaced, and after having made strong extension I dressed the limb with gutta percha splints, and laid it upon its side upon a pillow.

Oct. 3, I examined the limb, the splints having been removed for some time. He had been going about upon crutches for about three or four weeks. The limb was a good deal swollen, but appeared perfectly straight; nor could I discover any shortening.

Case LIII.—*Simple fracture. Result perfect.*

Thomas Healey, æt. 14, was struck by a cart wheel, breaking both bones of his left leg, through their lower third. Fracture simple and transverse; the doctor said it was "broken square off, like a piece of candy."

Dr. Peacock, of Cathron, Ireland, dressed the limb. He used side splints made of tin.

Twenty-one years after, I could not discover any trace of the fracture.

Case LIV.—*Simple fracture. Result perfect.*

Thomas Hazard, aged about twenty-five years, fractured both bones of the leg near the upper end of the lower third, Sept. 18, 1850. Simple. Fracture of tibia slightly oblique. Produced by the fall of a tree directly across the limb.

Dr. Sprague examined the limb on the eighth day, and dressed it with a starch bandage. There was then no displacement.

Oct. 1, he was received into the Hospital of Charity, at Buffalo, and on the third of October I renewed the starch dressing.

On the twenty-eighth day the bones were united; slight provisional callus. On the thirty-second day all dressings were removed. The limb was neither shortened nor deformed in any degree.

Case LV.—*Simple fracture. Result perfect.*

John Jarvis, of Port Robinson, C. W., broke the tibia and fibula near the upper end of the lower third, in Nov., 1851. The limb was treated with simple side splints, by Dr. King, of Port Robinson.

Six months after the accident, while he was an inmate of the Buffalo Hospital, I examined the limb, and found no shortening or deformity, and only a very slight amount of provisional callus.

Case LVI.—*Simple fracture. Result perfect.*

John H. Messersmith, æt. 15, fractured the tibia and fibula through the lower third, about four inches above the ankle-joint. Fracture produced by the passage of a loaded wagon across the leg. Simple.

Drs. Pride and Van Pelt, of Erie county, in attendance.

Six weeks after the accident I found the bones united without shortening, but surrounded with ensheathing callus. There was also a slight outward projection of the fragments.

Case LVII.—*Simple, oblique fracture. Result perfect.*

Angelica Lamb, æt. 7, fell, June 22, 1841, and broke both bones near their lower ends: oblique.

Five hours after the accident I dressed the limb with compresses, a pasteboard splint, and a roller, laying the limb upon a double inclined plane.

In four weeks union was complete, and with no displacement or deformity, except that a small ensheathing callus remained. All dressings were at this time removed, and the patient permitted to use crutches.

CASE LVIII.— *Simple, transverse fracture. Result perfect.*

July 14, 1846, I was requested by John Trowbridge, to see with him a woman whose leg had been broken on the last day of June. She was traveling. Much swelling had supervened and no attempt had yet been made to restore the fragments to place. We found the displacement lateral, and easily overcome. Straw junks were employed as lateral supports.

The bones united without shortening or deformity.

CASE LIX.— *Simple fracture. Result perfect.*

John Keef, æt. 17, was thrown from a wagon, breaking his leg and his lower jaw at the same time. (See Case VI, Inf. Max.) The leg was broken near the upper end of the lower third. Fracture of the tibia oblique.

I dressed the leg with gutta percha splints. The bones united without shortening and with only a very slight bend at the seat of fracture.

CASE LX.— *Simple fracture. Result perfect.*

Joseph Henry, æt. 6, broke his leg about two inches above the ankle. Fracture simple.

Dr. Charles Wilcox was employed.

Four months after I found the limb in every respect perfect.

CASE LXI.— *Simple fracture. Result imperfect.*

O. C. Martin, æt. 40, was admitted to the Buffalo Hospital of the Sisters of Charity, Dec. 6, 1856. On the evening preceding he had fallen through a hole in a plank sidewalk, and broken his left leg. It was dressed by Dr. N., of Buffalo, with two lateral splints, binders board, rollers, &c.

I found the limb much swollen and vesicated, with a purple spot upon the internal malleolus produced by the pressure of the splint. Fracture in the tibia three inches and three-quarters from the lower end of the malleolus internus; oblique; direction of obliquity from before backward and downward; the upper fragment in front of the lower. Limb shortened half an inch. The exact point of fracture in the fibula we could not determine.

Dr. Lemon and Mr. Coventry made extension and counter-extension, with the limb flexed, but they were unable, even for one moment, to extend the limb to the same length with the other.

I then applied two gutta percha splints, which enveloped nearly the whole limb, and laid his leg upon its outside, after the manner of Pott.

Dec. 31. The fragments were firm, and I substituted the paste bandage for the gutta percha. On the following day he left his bed.

Jan. 10, 1857, he was dismissed from the hospital. The limb seemed perfect except that it was shortened rather less than half an inch.

CASE LXII.—*Simple fracture. Result imperfect.*

Michael Dolen, æt. 26, broke his left leg by a fall, May 15, 1853. Fracture of both bones three inches above the ankle.

Drs. Pratt and Garvin were at first employed. A splint was secured to each side of the limb, and the leg was then laid upon a pillow, upon its back.

At the end of two weeks he was sent to the Hospital of the Sisters of Charity, and was placed in Dr. Winne's ward.

April 7, 1854, nearly one year after the accident, Dolen consulted me, at my office. The limb was shortened half an inch. The lower end of the upper fragment projected in front three-quarters of an inch, and the lower part of the leg and foot had fallen back out of line about 10°. It was also slightly inclined to the fibular side. The fibula was thrown toward the tibia at the seat of fracture. The leg was as useful as before, except that he could not run as fast.

CASE LXIII.—*Simple fracture. Result imperfect.*

John Granger, of Hungerford, England, æt. 43, was tripped by a stone while walking, breaking his right leg through its lower third. Fracture simple and oblique. It was treated by Richard Barker, surgeon, of Hungerford, England. He employed only side splints.

Two years after, I found the leg shortened one inch, the upper fragment riding upon the front and inner side of the lower fragment.

CASE LXIV.—*Simple fracture. Result imperfect.*

Mrs. Hoyt, of Canaan, Conn., æt. 33, fractured her left leg through its lower third. The limb was treated by Dr. Plumb, of the same place.

Forty years after the accident occurred, Oct. 28, 1852, I found the leg shortened half an inch, and the whole limb, below the fracture, much bent inward. The lower end of the upper fragment projecting, also on the inside of the lower fragment. The ankle has always remained quite stiff.

Mrs. Hoyt says the reason why the bones are not in place is, that "the fracture was oblique, and the doctor could not keep it in place, although he set it three times."

CASE LXV.—*Simple fracture. Result imperfect.*

Dr. A. G. Bigelow, of Rochester, N. Y., æt. 22, was thrown from a sleigh breaking both bones of his left leg through their lower thirds. Fracture simple and oblique. Dr. A. Goldsmith, of Rochester, and now of New York, was employed.

Ten years after, I found the leg shortened half an inch, and to compensate for this he wore an extra piece of leather under his heel. There was also a slight projection at the seat of fracture. There was no other imperfection in the limb.

CASE LXVI.—*Simple fracture. Result imperfect.*

John Graefe, of Buffalo, æt. 30. A heavy board fell edgewise upon his right leg, Jan. 12, 1856, breaking both bones obliquely, about four inches above the joint. The fragments were displaced, and the lower end of the upper fragment of the tibia was thrown to the fibular side of the lower fragment. It was not carried forward.

I made powerful efforts, by extension and counter-extension, to bring the fragments into line, but I was unable to do so. I then dressed the limb with a couple of Welch's veneered splints, having carefully padded them, and laid the limb upon its fibular side upon a pillow; the leg being flexed upon the thigh.

Jan. 19, assisted by Dr. Baker, I removed and reäpplied the dressings. From this time until the cure was completed, the limb was very attentively watched, and as often as the bandages became loosened they were tightened or renewed. In five weeks bony union was complete and the splints were removed, except that they were to be reäpplied at night for a week or two longer. Soon after this he began to use crutches. The usual swelling of the limb occurred, and his ankle remained stiff for several months, no doubt from the effect of the fracture upon the muscles, for the ankle was never inflamed. The limb was shortened less than half an inch, and the lateral displacement was very inconsiderable.

Such was the condition of his limb when I learned from my friends of the profession in the city, that he was inquiring of them whether he ought not to obtain damages for bad surgery. It is most likely, also, that I am indebted to their honorable conduct for having escaped a prosecution, where I believed I had merited a compliment.

Case LXVII.—*Simple fracture. Result nearly perfect.*

Peter Best, of Buffalo, æt. 31. His left leg was caught between his horse and a load of wood, breaking both bones three and a half inches above the joint. Dr. Lay was called, and laid the limb on Welch's double inclined plane, applying also lateral splints.

March 1, 1856, the sixth day after the accident occurred, Dr. Lay requested me to see the limb with him. It was very neatly and appropriately dressed, and the fragments, having never been much displaced, were now, also, in very nice apposition. The lower end of the upper fragment was, however, slightly overriding the lower fragment, and projecting about three lines.

This treatment was continued, and the fragments united. The bones were pretty firm on the 19th of March, (24th day,) and very little, if any, ensheathing callus could be felt. On the 28th, the starch bandage was applied.

April 18th, he began to walk with crutches, but complained very much of his ankle, as being both painful and stiff. This stiffness continued a long time. The limb is shortened one quarter of an inch, and the fragments remain as when I first saw it. He walks now without any halt.

Case LXVIII.—*Simple fracture. Result imperfect.*

Wm. A. Hart, of Ct., æt. 15, broke the left tibia and fibula, about four inches above the ankle-joint. The fracture was simple, and the tibia was broken obliquely.

The limb was treated by Dr. Beecher, of Ct.

Oct. 1, 1856, forty-four years after the accident occurred, I find the lower end of the upper fragment of the tibia in front and to the inside of the lower fragment. The displacement in this direction is, however, slight. He says that his leg has shortened a little, perhaps one quarter of an inch. I cannot now ascertain, as the other leg has been broken also, and is shortened a little.

Case LXIX.— *Simple fracture. Result imperfect.*

Amos A. Henly, æt. 82. Simple fracture of both bones, about three or four inches above the ankle-joint. Dr. Lauderdale, of Geneseo, N. Y., was employed.

After one year I found the limb shortened half an inch.

Case LXX.— *Simple fracture. Result nearly perfect.*

James Alden, æt. 25; sailor; while wrestling, Jan. 20, 1856, he broke the left leg near the upper end of the lower third. The skin was wounded, but the wound did not communicate with the fracture.

Dr. Wilcox dressed the fracture, and then sent him to the Marine Hospital.

Jan. 22, Dr. Wilcox, as an act of courtesy to me, and that the students of the college might avail themselves of the instruction which the case afforded, requested me to dress the limb. I could feel a very slight jutting of the fragments at the place of fracture. I applied two broad and very carefully padded lateral splints, which I secured in place with a roller.

On the thirty-second day bony union had occurred, with a shortening of one-sixth of an inch. No deformity. No ensheathing callus.

Case LXXI.— *Simple fracture. Result imperfect.*

Thomas Flinn, æt. 38. Simple fracture, about three or four inches above the ankle. Dr. Frost, of Medina, N. Y., was employed as the surgeon.

Two years after the accident, I found the limb shortened one inch and a quarter.

Case LXXII.—*Simple fracture. Result imperfect.*

Thomas Shannon, æt. 40. Simple fracture near the upper end of the lower third. Dr. Havens, of Buffalo, was employed.

Eight weeks after the accident, May 21, 1850, Shannon called upon me complaining of his treatment by Dr. Havens. The limb was shortened half an inch and some bent at the seat of fracture.

Case LXXIII.—*Simple fracture. Result imperfect.*

Mrs. Maria Hatchley, æt. 54. Simple fracture, about two inches above the ankle. When I saw her she was under treatment at the New York Hospital. By the politeness of the house surgeon I was permitted to examine the limb. The bones were united with a shortening of one quarter of an inch.

Case LXXIV.—*Simple fracture. Result imperfect.*

Hemon Miller, æt. 43, fractured the leg about two inches above the ankle. Dr. Beckwith and Prof. James Webster, of Rochester, in attendance.

Ten weeks after the accident, Miller called upon me. The bones were united, but the callus was not firm. The limb was shortened half an inch.

Case LXXV.—*Simple fracture. Result imperfect.*

Jonathan Barker, æt. 33, fractured both bones, about two or three inches above the ankle. Fracture simple. Drs. Peter and Abraham Hogaboone, of Castleton, took charge of the case.

Thirty years after, I found the limb not shortened nor deformed, but its muscles were atrophied, and the ankle still remained stiff and tender.

Case LXXVI.—*Simple fracture. Result imperfect.*

John H. Landon, æt. 52. Dr. Josier, of York, Livingstone Co., N. Y., was employed.

Twenty-five years after the accident, I examined the limb. The leg is shortened half an inch. The fragments are united at an angle, the foot and lower part of the leg being inclined to the tibial side; ankle very stiff; enlarged; pains him very much, and more and more every year. His habits are temperate.

CASE LXXVII.—*Simple fracture. Result imperfect.*

Elijah Brewster, of Buffalo, æt. 42, fractured his right leg about three inches above the ankle joint. Fracture simple. Dr. James P. White in attendance.

Eight years after, I found the leg shortened one quarter of an inch.

CASE LXXVIII.—*Simple fracture. Result imperfect.*

Elijah Brewster, æt. 50, (same patient as Case LXXVII,) broke the left leg about two inches above the ankle.

Dr. Barrett first dressed the limb, but on the second or third day, Dr. Barrett requested me to take charge of it.

The limb was united in the usual time, and at the end of three months he was dismissed with a shortening of one-quarter of an inch.

CASE LXXIX.—*Simple fracture. Result imperfect.*

Ed. M'Donald, of Buffalo, aged about 35 years, broke his leg by the fall of a tree upon it in 1852. Fracture at the upper end of the lower third. Simple. He was taken to the Hospital of the Sisters of Charity.

Dr. Winne in attendance.

Six weeks after the accident occurred, the fragments were united with a shortening of three-quarters of an inch. The upper fragment of the tibia lies in front and to the tibial side of the lower fragment.

CASE LXXX.—*Simple fracture. Result imperfect.*

Alvin Vanderhyden, æt. 37, fractured the tibia and fibula about two or three inches from the ankle-joint. Simple.

Dr. Jakeway, of Cayuga Co., N. Y., treated the fracture.

Twenty-five years after, I found the leg shortened half an inch, and the foot, with the lower part of the leg, bent inward very much.

CASE LXXXI. — *Simple fracture. Result imperfect.*

Justice Hubbard, of Hamburg, Erie Co., N. Y., æt. 40, had his right leg broken by the fall of a tree. Tibia broken obliquely about two inches above its lower end, and fibula about two inches higher.

The limb was dressed within ten hours after the accident, by Dr. Camp, of Hamburg. It was already much swollen. Two or three men made extension and counter-extension. Suitable bandages, with three lateral splints, were applied. The splints were made of shingles and binders' board. It was then laid extended upon the bed, without any farther dressing, except what might be necessary to support it in position.

After eight years I examined the limb. He says that its perfect use was not recovered under six months, since which time he has experienced no lameness or inconvenience. The leg is shortened half an inch. The lower end of the upper fragment of the tibia is displaced inward one quarter of an inch. The lower end of the upper fragment of the fibula is displaced backward. A large amount of bony callus remains upon the tibia.

CASE LXXXII. — *Simple fracture. Result imperfect.*

—— Howard, of Herkimer Co., N. Y., æt. 50, broke the tibia of the right leg at the upper end of the lower third. Fracture oblique. At the same time, also, the fibula was broken three inches above the ankle.

The limb was dressed and subsequently treated by Dr. Doolittle, a distinguished surgeon in Herkimer Co.

Eighteen years after the accident, I find no perceptible shortening of the limb, but there remains a slight lateral displacement of both bones, at the seat of fracture, and also a backward displacement, by which the foot is thrown a little forward. During several years he was a little lame, but all lameness has now disappeared.

CASE LXXXIII. — *Simple fracture. Result uncertain.*

June 29, 1848, —— Humphreyville, æt. 85, broke his left leg just above the ankle. Fracture oblique.

I was called soon after the fracture occurred. Assisted by my pupil, Stephen Smith, I reduced the fragments, and having secured them in place with two lateral splints, I laid the limb over a Potter's double inclined plane.

Four months after the limb was firm, and I think without shortening or deformity; I measured the limbs, but I have no record of the fact.

Case LXXXIV.—*Simple fracture. Result imperfect.*

Asahel Turtelot, æt. 49, broke his left leg near the upper end of the lower third. Fracture simple.

Drs. Lap and Fuller, of Lowderdale, Ohio, were employed.

Two years after the fracture had occurred, Turtelot called upon me for counsel, intimating that he intended to sue his surgeons for damages.

I found the limb shortened three-quarters of an inch, and still swollen. I could not discover any error in the treatment to which these results were properly chargeable.

Case LXXXV.—*Simple fracture. Result imperfect.*

Ed. McDonald, about 35 years old, broke the tibia and fibula near the upper end of the lower third, in 1852. On the following day he was admitted to the Hospital of the Sisters of Charity. Dr. Charles Winne's service. The fracture was simple and oblique.

Six weeks after the fracture occurred the bones were united, with a shortening of three-quarters of an inch. The upper fragment overrides the lower, it projects also somewhat to the tibial side.

The limb has been treated with lateral splints, well padded, and without extension, except what could be accomplished through these side splints.

Case LXXXVI.—*Simple fracture. Union delayed, &c.*

Wm. Crabbe, of Burford, C. W., æt. 47. Mr. Crabbe slipped and fell upon an icy sidewalk, in Jan. 1856, and his right leg coming under his body as he fell, was broken three inches above the ankle. Fracture simple. The tibia was broken obliquely, the direction of the fracture being downward and inward. Mr. Crabbe weighed at the time, about 240lbs.

Henry Ross, surgeon, of Burford, Canada, was employed. The limb was dressed with splints made of shingles and underlaid with cotton batting, and it was then placed on a pillow. During the following few days he thinks the inner splint hurt him very much. On about the sixth day the limb was laid over a double inclined plane, and now the heel became very painful, of which pain he was afterward somewhat relieved by lowering the knee.

Eleven weeks after the accident occurred, all dressings were removed.

Aug. 28, 1856, Mr. Crabbe consulted me at my residence in Buffalo. I found the leg shortened three-quarters of an inch, the lower end of the upper fragment being in front of the lower. The lower fragment inclined backward from the point of fracture down to the foot. The lower part of the limb remained swollen. He was unable to bear his weight upon it.

Mr. C. called my attention especially to what he supposed to be a motion between the fragments of the tibia, and which I at first also supposed did exist; but a more careful examination led me to think that it did not exist; indeed he told me himself that one side of the tibia was firm, but the other moved, a condition of things which would not be possible. If there was any motion between the fragments it was very little.

I advised him to discontinue all bandaging, and to bathe the limb in cold water.

Case LXXXVII.—*Simple fracture. Cured.*

Edward Hutchinson, æt. 27, admitted to the Hospital of the Sisters of Charity, Feb. 8, 1851. Left leg broken just above the ankle. Oblique. Fracture dressed by Dr. Sprague before admission.

I found the limb swollen, and having loosened the dressings I applied straw splints and laid the leg and thigh over a double inclined plane.

On the 24th of Feb. he was transferred to the Marine ward under the care of Dr. Wyckoff. Union took place in about six weeks. Of the precise character of the cure I am uncertain.

Case LXXXVIII.—*Simple fracture. Result imperfect.*

Capt. Thompson, of Buffalo, æt. 30, fell on board his vessel, July 14, 1855, breaking his right leg about four inches above the ankle. Fracture simple and oblique.

Dr. Brainard, of Chicago, first dressed the limb, with a splint fitted to the back of the leg. The leg was allowed to rest upon its back. On the third week he came under my care. I found the bones, Aug. 5th, (twenty-first day,) united, but with a slight overlapping and bend. I dismissed him at the end of about four weeks more, with the limb in the same condition, as to length, &c., as when I first saw him.

CASE LXXXIX. — *Simple fracture. Result imperfect. Prosecution.*

Joseph Hampshire, of Livingstone Co., N. Y., aged about 33 years, fractured the right leg two inches above the ankle-joint, sometime near the last of May, 1850. Fracture simple and nearly transverse. The soft parts were much bruised and the limb soon became excessively swollen.

Dr. Wm. H. Reynale, of Dansville, made the first dressing, assisted by Drs. Endress and Blake. The dressings applied were an eighteen tailed bandage, pasteboard splints, and Hutchinson's splints. On the following day Dr. Endress took charge of the patient, and continued the same dressings until the tenth day, when he substituted for Hutchinson's splints, "Jarvis' adjuster." On the sixteenth or seventeenth day, the limb showing a constant inclination to rotate outward, it was again carefully brought into position. No union of the fragments had occurred. Drs. J. L. Endress and Patchen were then in attendance. On the twentieth day Dr. Reynale resumed his charge of the patient, and continued in charge until the close of the treatment. It resulted in a union, after much delay and suffering. A deep ulcer on the back of the heel.

In April, 1852, about two years after the accident occurred, I examined the leg.

The bones were united without shortening, but with a slight deformity; the leg being bent laterally from the point of fracture. I could not see that he limped when walking, and he seemed to step firmly and freely. I was told, however, that he trod a little upon the outside of his foot.

Hampshire subsequently sued both Drs. Endress and Patchen for damages, and the case was tried, I think, in the fall of 1852, at the Livingstone County Circuit, held at Geneseo, the Hon. Judge Selden, presiding. On the part of the defendants, Messrs. Hastings, Skinner and Endress, were employed as counsel; and on the part of the plaintiff, Messrs. Wisner and Peck.

The jury were unable to agree and were discharged. The case was never again brought to trial.

(To be Continued.)

